# Definition of character for medical education based on expert opinions in Korea

**DOI:** 10.3352/jeehp.2021.18.26

**Published:** 2021-09-29

**Authors:** Yera Hur

**Affiliations:** Institute of Medical Education, College of Medicine, Hallym University, Chuncheon, Korea; Hallym University, Korea

**Keywords:** Character, Medical education, Humanities, Professionalism, Medical student

## Abstract

**Purpose:**

This follow-up study focused on 3 overarching questions: what keywords can be extracted from experts’ definitions of character?; what is the operational definition of character for medical students?; and what possible solutions can be suggested to address the issues of character education that were identified in the previous study?

**Methods:**

Sixty-three medical education experts recruited through expert sampling and 19 non-medical education experts recruited through snowball sampling answered a questionnaire that addressed the 3 major questions of the study. The responses were analyzed for descriptive statistics with supplementary keyword extraction tools, including the Cortical and Monkey keyword extractors.

**Results:**

A total of 93 definitional statements were counted, and 138 keyword terms were extracted. The top 5 keyword terms mentioned by the medical education experts were “patient”, “empathy”, “qualities”, “attitude”, and “ability”. These keyword terms were quite different from those mentioned by the non-medical education experts. Based on the extracted keywords, an operational definition of character education by the medical education expert group was presented as follows: the basic qualities and ability to empathize with patients affected by illness based on respect for patients and others. Various methods were proposed to solve the issue of character education, and many of them pointed to curriculum development, such as improvements in teaching and learning methods and evaluation methods, including role modeling.

**Conclusion:**

A clear statement of the concept of character education is the start to resolve issues of character education. Character education improvements will be possible at the institutional level according to the above results.

## Introduction

### Background

Issues regarding physicians’ medical students’ character and professionalism have been discussed in the media for decades [1-3] and are an urgent issue that we must face directly. As medical educators, going back to the basics means reflecting on whether we are doing the best possible job of educating and training “good doctors”. The first step in doing this is to identify what a good doctor is. This concept has been discussed for many years, and we now have some clear definitions of good doctors specific to our current society [4,5].

The next step is to identify the education needed to educate our medical students to become “good doctors”. This process could involve many aspects and content of medical education, and medical professionalism emerged as an important keyword in medical education along with medical humanities [6,7]. Responding to this trend, all medical schools in Korea have organized and emphasized the development of medical education or medical humanities departments and curricula during the last 2 decades [8]. Despite these efforts, the issues of character and medical professionalism have not fully subsided.

Character education is an essential foundation for the rest of medical education. Previous studies have focused on identifying the core elements of character education [6,7]. Therefore, it would be indispensable to gather opinions from medical education experts on how they conceptualize character education.

### Objectives

This is a follow-up study of Hur and Lee’s previous studies [6,7]. It addressed the core elements of character education for medical students and the issues of character education in medical education.

This study focused on the following overarching research questions: What keywords can be extracted from the experts’ definitions of character?; What is the operational definition of character for medical students?; What possible solutions can be suggested to address the issues of character education that were identified in a previous study?

## Methods

### Ethics statement

This study was approved by the Institutional Review Board of Hallym University (HIRB-2018-049-2-CC). Informed consent was obtained from the subjects.

### Study design

This is a survey-based content analysis study conducted by extracting keywords and developing an operational definition of character education.

### Setting

A single questionnaire with 3 major questions on character was distributed to medical education experts in Korea via e-mail. The questions were: “How would you define the ‘character’ that is required from a good doctor in the era of the fourth industrial revolution?”, “What are the issues of character education in current medical education (if any?)”, and “If you agree that there are any issue(s) of character education in current medical education, what possible solutions do you suggest?” The survey was distributed twice.

In the first round, 145 e-mails were sent, and the answers were collected from September to October 2018. The low response rate from the medical education experts was expected due to their busy schedules. The second round of distribution was done the next year for non-respondents, and 29 additional responses were gathered from September to October 2019. The questionnaire surveys were completed in paper and pencil offline or online according to the respondents’ preferences.

### Participants (subjects)

Expert sampling was chosen for the medical education experts due to the nature of the question, and the results may not necessarily be generalizable to the entire population. It is known that combining expert sampling with an additional sample of non-medical education experts enhances the reliability of the data. For this purpose, snowball sampling was used to recruit non-medical education experts who were willing to answer the survey questions. This group consisted of 2 nursing education experts, 11 private practice physicians, and 6 medical students.

The expert sampling method was used to gather experts’ definitions on character education for doctors. The list of medical education experts was drawn from the medical education departments of all 40 medical schools in Korea. The medical education experts were required to be the person in charge or affiliated with the medical education department of their respective medical school, or to have at least 5 years of experience in medical education. They were required to have an affiliation with a committee or institution that was representative in relation to medical education (Table 1). For the medical education specialists affiliated with medical schools, their medical education-related departmental work experience and student education experience were investigated as primary data. The average length of student education experience was 19.3 years (minimum, 3 years; maximum, 40 years), and that of related working experience was 12.5 years (minimum, 1 year; maximum, 35 years).

In the first round of the survey, 145 e-mails were sent, and the response rate was 23.4% (34 responses). In the second round of survey distribution, 29 additional responses were gathered. Thus, responses from 63 medical education experts from 30 medical schools or colleges and 19 non-medical education experts were used in the final analysis.

### Data sources/measurement

Since the operational definition of “character” cannot be measured, the keyword extraction method was used. Keywords were extracted separately from the medical education experts’ definitions or statements on character education and the statements made by the non-medical education expert group and combined. As supplementary keyword extraction tools, the Cortical and Monkey keyword extractors played an auxiliary role in decision-making [9,10]. The definitions and statements were entered into the keyword extraction programs, and the automatically extracted keyword terms were organized in an Excel sheet (Microsoft Corp., Redmond, WA, USA)(Dataset 1).

### Statistical methods

As the extraction results of the Cortical and Monkey programs were not perfect, the relevance of the keywords obtained from the 2 keyword extraction programs were analyzed one by one and the keywords were added and subtracted from the existing terms [9,10]. The extracted keywords were grouped with similar concepts to describe the final concept of character education.

## Results

### Extracted keywords related to character

From the concepts described by a total of 82 respondents, 93 statements were counted. When very similar expressions were grouped, a total of 138 keyword terms were extracted. These 138 words were mentioned at least once and at most 39 times, as shown in Table 2.

**Fig. 1.**
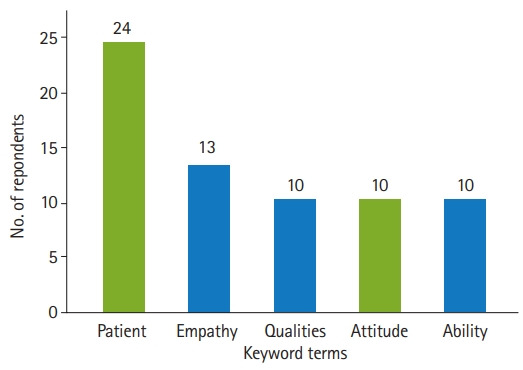
Top 5 keyword terms related to character mentioned by medical education experts (n=63).

Thirty-one keyword terms, such as “commit”, were mentioned twice, and 68 terms, such as “collaborate”, were mentioned once. Table 3 shows the terms mentioned 3 or more times by the respondents. The top 5 keyword terms mentioned by the medical experts were “patient” (38.1%), “empathy” (20.6%), “qualities” (15.9%), “attitude” (15.9%), and “ability” (15.9%). The top 5 keyword terms mentioned by practicing physicians, medical students, and nursing school professors were “patient” (78.9%), “understanding” (47.4%), “attitude” (26.3%), “heart” (26.3%), “life” (21.1%), and “point of view” (21.1%). The 2 terms in yellow bars in Fig. 1, “patient” and “attitude”, were the most common terms mentioned in both respondent groups (Table 3, Fig. 1).

### The operational definition of character for medical students

Including the top 10 keyword terms (patient, qualities, ability, attitude, respect, others, human being, ethics, responsibility, communication, cooperation, colleagues) mentioned by the medical education experts, the following operational definition of character for medical students was developed:

The basic qualities and ability to empathize with patients affected by illness based on respect for patients and others, to have basic ethical awareness and responsibility for human life, and to cooperate and communicate with colleagues.

The top 10 keyword terms mentioned by non-experts were “patient”, “empathy”, “qualities”, “understanding”, “attitude”, “human being”, “ability”, “ethics”, “others”, and “life”. With these terms, the following operational definition of character for medical students was developed:

The basic qualities, attitude, and ability to understand and empathize with patients and others, to have basic ethical awareness.

### Solutions to the issues of character education

In the previous study [7], the respondents pointed out some issues related to character education in Korea. A considerable number of responses pointed out that despite notable curriculum reform movements towards skill and practice-based systems along with an emphasis on medical professionalism and humanities, many medical schools and colleges still have a knowledge-oriented educational system. There is also a lack of interest in the evaluation of character education, insufficient research on the content of character education, and a lack of character education awareness among both education providers (professors, administrators) and consumers (students). A possible explanation for the culture and real-world circumstances where academic performance matters the most may be that stakeholders do not value character education as much as they should. Hur and Lee [7] also found that medical schools did not have sufficient teaching human resources to handle character education. Some comments emphasized that the concept of character education is ambiguous, which is one of the primary reasons for this follow-up research.

**Fig. 2.**
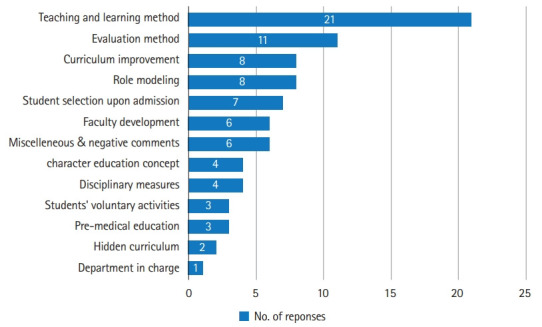
Type of solutions to character education issues divided into categories.

Forty-six medical experts, 2 physicians, and 1 nursing school professor responded to the question, where duplicate responses were possible. Eighty-four statements or ideas were suggested as the solutions to the issues of character education (Supplement 1). The responses were categorized, and numerous answers were on the subject of teaching and learning methods of character education (Fig. 2). Some ideas were given by the respondents.

“For character education, field education, including clinical practice, is a more effective method than lectures, but I think that the field of character education practice in Korean medical schools is not educational at all.”
“If flipped learning is actively introduced so that students can help each other in class and create results, that in itself would be very helpful for character education.”
“Reinforcing team-based activity and reflecting peer evaluation, and writing essays on a variety of topics.”
“It is necessary to organize the content and format of the medical humanities curriculum in such a way that experience is possible. It is necessary to have a program that exposes students to real situations rather than lectures. Programs should provide opportunities to experience empathy, consideration, and dedication, as well as a system to reflect on them. For example, after pre-medical years, a one-to-one relationship with an elderly patient can be established. In addition to regular visits, a program can be operated to participate in medical care together during hospital visits until graduation. Debriefing opportunities should be provided to reflect on the results.”

The responses were followed by some ideas on evaluation methods, role modeling issues, and curriculum development.

[Responses on the evaluation method]
“A portfolio including self-reflection for each item of personality element and feedback on it (multiple evaluations are required, and the evaluator must receive proper training before proceeding with the assessment).”
“Reinforcing team-based activity and reflecting peer evaluation, and writing essays on a variety of topics.”

[Responses on role modeling]
“It is necessary to change the character quality of the self-proclaimed medical education executives (those who are assigned in such positions). We need people with the humility to know that what they know is not everything and respect other fields of study. Doctors don’t know how ignorant they are. The crux of the matter is that they want to spend their whole lives with the triumph of their teenage years. You reap what you sow.”
“In the end, the appearance of senior doctors/professors who see and hear what happens in the clinical field determines the success or failure of character education as a whole.”

[Responses on curriculum development in general]
“Recently, unlike in the past, students’ character qualities show individual, selfish, and hypocritical behaviors, so it is urgent to develop a curriculum and professors specializing in character education.”
“If possible, we should apply interprofessional education to teaching/training not only within the medical school context/curriculum, but also with students in related fields such as nursing, clinical psychology, pharmacy, and social work. I am sure that this will be a good opportunity for dialogue between occupations, exploration, self-discovery, and understanding other occupations. It is hoped that this IPE model will be introduced and expanded in many medical schools in Korea.”

There were some notable suggestions in the student selection category as well.

“It seems necessary to find a way to choose well when selecting incoming students. The 4–6 years of education after admission may help, but the basic tendencies also seem to be necessary.”
“Within the current system, I think the most specific alternative is to develop a method for selecting students with outstanding character at the time of admission.”

Some responses dealt with the hidden curriculum and the need for faculty development, including some negative responses saying that character building is home education, not medical education. There were also doubts about whether character education can be taught and evaluated properly (Fig. 2).

## Discussion

### Key results

This study was a follow-up of the previous studies, which established the necessity and core elements of character education and the problems of current character education. In this study, the keywords were extracted from respondents’ definitions of character education, and an operational definition of character education was developed.

This study gave some suggestions on how to solve those issues of character education brought up in the previous study. From the above results, it is notable that some terms highly mentioned by the medical education experts, such as “empathy”, “qualities”, and “attitude” were not frequently mentioned by other respondents. Conversely, terms such as “understand”, “heart”, and “point of view” had a higher count for other respondents than for the medical education experts.

The top 5 keyword terms mentioned by the non-medical education experts were “patient” (78.9%), “understand” (47.4%), “attitude” (26.3%), “heart” (21.1%), “life” (21.1%), and “point of view” (21.1%), which were quite different from the medical experts’ choices. This means that the definition or concept of character education may differ to some extent between medical education experts and other health personnel.

### Interpretation

Different groups can have different perceptions on the subject of character education; therefore, the results should be considered when developing new curricula or setting goals in medical education [11,12]. The definition of character given from the previous study is as follows [7]:

“The character that a doctor requires is the basic attitude, values, and mindset that must be present to perform his or her duties. These include respect for human beings, empathy and consideration for patients, a sense of calling, honesty, ethics, and responsibility.”

This definition of a doctor’s character was drawn from the core elements identified by the Delphi survey in the research. The keywords in this statement were “respect for human beings”, “empathy”, “consideration”, “patients”, “calling”, “honesty”, “ethics”, and “responsibility”. If readers look at the keywords of the concepts stated above and the keywords of the concepts stated in the operational definition of character in this study, it can be seen that many words are identical or similar, as shown below.

Education that fosters the basic qualities and ability to ***empathize*** with ***patients*** affected by illness based on ***respect*** for ***patients*** and others, to have basic ***ethical*** awareness and ***responsibility*** for ***human life***, and to cooperate and communicate with colleagues.

Respondents gave diverse ideas for solutions, but many thought that instructional methods should be reformed or need improvement along with changes in the evaluation method of character education, which go side by side in any curriculum development initiative. Some answered in broad terms that there needs to be some kind of curriculum improvement. Others specifically pointed out that character education should be started in pre-medical education, not only in teaching and learning methods and evaluations in medical schools. Therefore, the majority of the responses dealt with the wide-ranging theme of curriculum improvement. Nonetheless, the experts did not overlook the importance of faculty development, which is an essential part of any curriculum development [13]. Role modeling and mentoring are also of substantial importance; in particular, role modeling could be the most potent teaching strategy in changing students' perceptions or developing attitudes or behaviors [14].

Solving these issues is very important to bring about real change. In this study, experts’ solutions to the character education issues were presented, and some concrete ideas were also included. One of them was that character education or evaluation should start from the admission stage and pre-medical education. Most medical schools and colleges in Korea have adopted aptitude and personality interviews as part of the medical school admission interview process (i.e., multiple mini-interviews) to evaluate the qualifications, character, and aptitude required to major in medicine. Opinions were also presented by the medical education experts and National Assembly Health and Welfare Committee audit. It may also be worth considering whether to evaluate personality or character during medical license examinations as these examinations are the last phase before becoming a medical professional [15,16]. However, according to the Research & Development report of Korea Health Personnel Licensing Examination Institute, due to the difficulty in ensuring the reliability and validity of character evaluation in an examination, the feasibility of implementing character evaluation in the medical licensing examination is very low [17]. Nevertheless, the author would like to strongly emphasize that strengthening character education in medical education is essential.

### Limitations

Medical education experts comprised the majority of the population (75%), whereas relatively few responses were obtained from the non-expert group (private practicing physicians, nursing professors, medical students) and the responses from the non-expert group are subject to the basic limitations of the snowball sampling method. Therefore, the results from the non-expert group may have limitations in terms of generalizability.

### Suggestions

Based on the concept and core elements of character education revealed in the results of this study and prior research, institutions can provide a framework of character education. The definition of character and some character education instructional ideas suggested by the experts from this study, along with the 8 core elements of character education from the previous study, can be used to provide a framework for character education in medical schools and colleges. A longitudinal framework of character education could be formed with specific examples of embedding the elements of character education in the curriculum by showing appropriate teaching and learning activities and assessment methods for each domain. Furthermore, apart from the medical education expert group’s or medical students’ point of view, a survey on the patient and families’ opinion of character education is valuable as it brings the medical consumer’s perspective.

### Conclusion

The definition of character education was derived from the keywords extracted from the medical experts. Starting with the statement of the concept of character education, some solutions could be found to the issues of character education. In particular, it is necessary to strengthen character education starting from the entrance exam and pre-medical education, which are the beginning stages of medical education. In addition, character education should be improved through consistent inclusion throughout the entire process of medical education. Methods for effectively learning and evaluating attitudes, such as field trips, practice-based cases, journals and logs, role model learning, and peer evaluation, rather than current lecture-style education should be incorporated. Based on the results of this study, it is expected that institutions will be able to find ways to improve character education.

## Figures and Tables

**Table 1.** Number of survey responses from each category of subjects

Subjects	Survey round^a)^	No. of responses
Medical education experts		
Medical school professors	1st	34
	2nd	29
Subtotal		63
Non-medical education experts		
Private practice physicians	1st	11
Medical students	1st	6
Nursing school professors	1st	2
Subtotal		19
Total		82

a) The first survey period: September to October 2018, the second survey period: September to October 2019.

**Table 2.** Number of mentions of the extracted keyword terms

No. of mentions	No. of keyword terms
1	68
2	31
3	7
4	6
5	4
6	2
7	5
8	4
9	1
10	1
11	4
12	1
14	1
15	1
16	1
39	1
Total	138

**Table 3.** Keyword terms related to character mentioned 3 or more times by respondents (N=82)

Rank	Keyword terms (no. of times mentioned)	No. of mentions by respondent groups		Total no. of mentions
		Medical education experts (n=63)	Others^a)^ (n=19)	
1	Patient/s (32), pain of the patient (2), patient’s pain (2), patient difficulties (1), patient care (1), life of patient (1)	24	15	39
2	Empathy (13), empathize (5), empathic (1), empathizing (1)	13	3	16
3	Qualities (8), basic qualities (1), internal quality (1), human quality (1)	10	5	15
	Attitude (8), professional attitude(s) (2), basic attitude (1), conscientious attitude (1), positive attitude (1), unchanging attitude (1)	10	2	13
	Ability (11)	10	1	11
6	Human being (5), humans (4), humane (1), human nature (1)	9	3	12
	Ethics (3), medical ethics (3), professional ethics (2), sense of ethics (1), ethical judgment (1), basic ethical consciousness (1)	9	2	11
	Others (11), help others (1)	9	2	11
9	Respect (8)	8	0	8
10	Responsibility (6), responsible (1)	7	0	7
	Communicate (3), communication(s) (2), skill to communicate (1), communication ability (1)	7	0	7
	Cooperation (5), community cooperation (2), cooperate (1)	7	1	8
	Colleagues (6), co-worker(s) (2)	7	1	8
14	Honesty (6), honest (1)	6	1	7
	Consideration (6), considerate (3)	6	3	9
	Life (4), human life (2), worthwhile life (1), life or death (1), healthier social life (1), love of life (1)	6	4	10
	Understand/ing (14), comprehensive understanding (1)	6	9	15
18	Caring (2), care (2), best possible care (1)	5	0	5
	Caregiver/s (6)	5	1	6
	People (6), loves people (1)	5	3	8
	Morality (5), moral character (1), moral influence (1)	5	3	8
22	Sympathy (3), emotionally sympathetic (1)	4	0	4
	Family (4)	4	0	4
	Value/s (4), clear values (1)	4	1	5
	Society (4), member of society (1)	4	2	6
26	Self-reflection (2), self-reflect (1)	3	0	3
	Desirable (2), desirable thought (1)	3	0	3
	Competence (1), medical professional competence (1), practical competence (1)	3	0	3
	Characters (1), basic character (1), characteristics (1), innate character (1)	3	1	3
	Altruism (4)	3	1	4
	Professional (3), profession (1), professionally (1)	3	2	5
	Heart (7), warm heart (1)	3	5	8
33	Role (2), given role (1)	2	1	3
	Relationship/s (1), healthy relationship (1), mutually good relationship (1)	2	1	3
	Service spirit (2), serve (1), service mind (1), spirit of service (1)	2	2	4
36	Compassion (2), compassionate (1)	1	3	4
	Sincerity (2), sincere (2)	1	3	4
	Point of view (4)	1	4	5
37	Treatment (2), applicable treatment (1)	0	3	3

a) Private practicing physicians, medical students, nursing school professor.

## Footnotes

**Data availability**
Data files are available from Harvard Dataverse: https://doi.org/10.7910/DVN/S5JLIB Dataset 1. The experts’ definitions of character and the results of keyword term extraction from the definitions.

## Supplementary materials

Data files are available from Harvard Dataverse: https://doi.org/10.7910/DVN/S5JLIB

Supplement 1. Solutions to the problems of character education.

Supplement 2. Audio recording of the abstract.
