# Supplementary material for: Definition of character for medical education based on expert opinions in Korea
Source: J Educ Eval Health Prof. 2021 Sep 29;18:26. doi: 10.3352/jeehp.2021.18.26 (PMC8577989; doi:10.3352/jeehp.2021.18.26)
Supplement: Supplementary file 2 — Supplement 1. Solutions to the problems of character education. [file jeehp-18-26-suppl1.docx]

Supplement 1. Solutions to the problems of character education

| **Student selection upon admission** | **No. of responses** |
| --- | --- |
| - Thorough character quality test at the time of admission (too few parts that can be changed to character education after admission). - It seems necessary to find a way to select well when selecting incoming students. The 4-6 years of education after admission may be of some help, but the basic tendencies also seem to be important. - Screening is required in the student selection process. - First of all, we have to select students well, we need to educate the selected students as experts, and as professors. - Improvement of the selection program for medical students: improvement of the practice of selecting students based on academic achievement test scores. - In the student selection process, personality is reflected as an important factor in selection. - The most specific alternative is to develop a method for selecting students with outstanding character at the time of admission. | 7 |
| **Development of character education competency / goal / concept / scope** |  |
| - Developing character education competency: to derive character-related behaviors that match the mission and intentions of the university. - Agreement on the scope of character education is needed. - First of all, I think the concept of character education should be clarified and approached. Many people think that character education is education at the top of the table, and there are many people who think that character education that has already been formed is inevitable. It seems that it is necessary to clarify what ‘character education’ is that can be accessed in universities, especially in medical schools, and the curriculum, educational methods, and evaluation methods should be developed accordingly. - Character education sets learning (performance) goals to improve awareness and behavior change rather than providing relevant knowledge, and introduces various teaching methods for this purpose, but it also focuses on external factors (class atmosphere according to grade level, social situation, etc.). Care should be taken so that pedagogical reflection can be reflected together. | 4 |
| **Department in charge** |  |
| - It is necessary to form a core professor group that can take charge of character education. | 1 |
| **Role modeling** |  |
| - Provide character-related faculty development programs so that professors can set an example in clinical practice. - It is necessary to change the character-quality of the self-proclaimed medical education executives (those who are assigned in such positions). We need people with the humility to know that what they know is not everything, and to respect other fields of study as well. Doctors don't know how ignorant they are. The crux of the matter is that there are so many arrogant people who want to spend their whole lives with the triumph of their teenage years. You reap what you sow. - In particular, in the case of clinical practice, it is very important to set the example of clinical practice professors. “How many role models does your school have?” - Seeking collaborative change with medical groups: role models. - The appearance of senior doctors/professors who see and hear in the clinical field determines the success or failure of character education as a whole. - As experts, and as professors, we need to do be good role models. - It is more important for character education to be learned through extracurricular activities rather than the regular curriculum or for professors to be good role models. Unlike a medical student, when he/she becomes a resident, I saw a lot of changes in character and attitude depending on the atmosphere of the department. - Teachers serve as role models with character so students can see and learn. | 8 |
| **Hidden curriculum** |  |
| - I think the key lies in the Hidden Curriculum. There is a limit to education through the curriculum at school. - Emphasis on hidden curriculum, providing various optional educational opportunities in addition to formal curriculum. | 2 |
| **Curriculum improvement** |  |
| - Excessive performance-oriented education should be avoided, and for this purpose, the number of hours of class should be reduced. And basic manners and ethics should be emphasized. - A more systematic professional consciousness education is required. - Adjust the education period - Character education is not limited to certain courses or classes. Continuing education is required in various class subjects. - In the pre-medical department, basic character education is implemented, and in each grade of the main department, character education is intensified step by step according to the stage of each curriculum. - Curriculum development according to the scope setting. - Recently, unlike the past, students' character qualities are showing individual, selfish, and hypocritical behaviors, so it is urgent to develop a curriculum and professors specialized in character education. - Should be reflected in the curriculum. It's boring, but most students don't seem to care if they don't reflect it on their grades in some way. - If possible, apply interprofessional education to teaching/training not only within the medical school context/curriculum, but also with students in related fields such as nursing, clinical psychology, pharmacy, and social work. I am sure that this will be a good opportunity for dialogue between occupations, exploration, self-discovery, and understanding other occupations. It is hoped that this IPE model will be introduced and expanded in many medical schools in Korea. | 8 |
| **Teaching & learning method** |  |
| - Introduction of instructional methods that can internalize direct and indirect experiences such as case-oriented role play and reflection - If flipped learning is actively introduced so that students can help each other in class and create results, I think that it will be of great help to character education by itself. - For character education, field education including clinical practice is the most effective than lectures, but I think that the field of character education practice in Korean medical schools is not educational at all. We will have to put more effort into developing the field of character education. Practitioners, nursing hospitals, and hospice wards should be more emphasized. - In hospital practice or clinical medicine courses, I hope that clinical subject professors will more actively include the aspect of character education in their educational content. - I would like to see more opportunities like the Grand Round in hospitals to increase student participation. - Self-directed timing, learn with cases, and experience. - It is necessary to organize the content and format of the medical humanities curriculum in such a way that actual experience is possible. It is necessary to have a program that is exposed to real situations rather than lectures. Provide opportunities to experience empathy, consideration, and dedication, as well as a system to reflect on them (For example, after pre-medical years, a one-to-one relationship with the elderly patient can be established, and in addition to regular visits, a program can be operated that allows them to participate in medical care together during hospital visits until graduation. Provides debriefing opportunities to reflect on results). - Educational method improvement: Avoid lecture-style education and expand student-led classes. - Education using a module that includes the clinical situation that is the goal of the study (including an educational guide that can differentiate between essential and additional, and have the ability to find out the good and the bad for each situation) - The degree of character education is a basic medical education course, and it is a clinical practice course rather than a basic medical course in the curriculum because field-oriented experiences can be more effective outside the theoretical education range within the classroom or within a group school frame. It is considered that it is necessary to adjust the education method, composition, and period so that it can be converted into a field-based education through experience in the field. - The so-called character education focuses on the pre-medical years level, and the main education (medical years level) focuses on the theoretical study. There is a need for more experiential education in medical level. - Suggestion of specific and realistic educational methods and evaluation methods and education on them. - Ultimately, repeated education and training (practice) are important. - After setting the scope, the education method is developed accordingly. - Character education sets learning (performance) goals to improve awareness and behavior change rather than providing relevant knowledge, and introduces various teaching methods for this purpose, but it also focuses on external factors (class atmosphere according to grade level, social situation, etc.). Care should be taken so that pedagogical reflection can be reflected together. - In my opinion, in order to develop a character, it is necessary to learn the process so that you can feel both physically and mentally. Therefore, I think that developing an educational method that can be learned in the character education course can be a solution. - I would like to propose to open and supervise a process to solve problems together through problem-based learning. - Character education is not limited to certain courses or classes. Continuing education is required in various class subjects. - Start with character education at the beginning of each subject (course). - Activation of cooperative classes such as TBL and PBL. - It is necessary to organize the content and format of the medical humanities curriculum in such a way that actual experience is possible. It is necessary to have a program that is exposed to real situations rather than lectures. Provide opportunities to experience empathy, consideration, and dedication, as well as a system to reflect on them (For example, when entering the main department, a one-to-one relationship with the elderly living alone is established, and in addition to regular visits, a program is operated that allows them to participate in medical care together during hospital visits until graduation. Provides debriefing opportunities to reflect on results). - Reinforcing team base activity and reflecting peer evaluation and writing essays on a variety of topics. - A process of self-reflection through an essay is required. - Better use of small group discussions or introspective portfolios with high student participation. - It is most realistic to focus on tasks that students can answer themselves rather than lecture classes. It is often necessary to devise a service system for this part, in many cases, replacing it with blood donation and solving the problem. - Through role-play in case situations such as conflict, rejection, criticism, fear, anxiety, and worry, not only count what kind of mind/ emotional state one is in, but also mapping the other party’s (patient/family/colleagues) expectations from ‘me’ as a doctor. - It is necessary to diversify the field experience in various medical environments (public medical field, general hospital, private hospital, medical service, nursing hospital, etc.) as a student and help them to cultivate the moral virtues that doctors should have in advance. - Rather than indoctrinated theoretical education, students make themselves feel the importance of character development through reading and various field experiences and fill in their lack of character by discussing them. - Systematic learning about the necessity and history of medical technology that developed along with the history of mankind, and the impact of social science | 21 |
| **Students' voluntary activities** |  |
| - Providing opportunities for students to voluntarily develop their character (consultation (mentoring), orientation sessions, etc.). - Students write their own character quality-related activities as a portfolio and evaluate them. - Educational method improvement: Avoid lecture-style education and expand student-led classes. | 3 |
| **Pre-medical Education** |  |
| - Normalization of pre-medical education is necessary. It is difficult to change a student's ethical attitudes or values ​​as the grade level progresses. In the pre-medical department, help is needed to develop a broader perspective and to have a self-identity as medical student. - Pre-medical education, self-directed timing, learn with cases, and experience - In the pre-medical department, basic character education is implemented, and in each grade of the main department, character education is intensified step by step according to the stage of each curriculum. | 3 |
| **Evaluation method** |  |
| - Reflect in the evaluation - Avoiding Competitive Peer Relationships: Absolute Evaluation needed. - At least 5 questions included in the case of a doctor's accident - as a case problem. - It would be good to have a sufficient understanding of character education and evaluation possibilities in character education for the professors so that it can be applied to student education. - If there is a personality problem, they will not be promoted. - Portfolio including self-reflection for each item of personality element and feedback on it (multiple evaluations are required, and the evaluator must receive proper training before proceeding with the evaluation) - Reinforcing team base activity and reflecting peer evaluation and writing essays on a variety of topics. - Suggestion of specific and realistic educational methods and evaluation methods and education on them. - Items that evaluate character quality through the curriculum and practice courses may be helpful. - After setting the scope, development of evaluation method accordingly. - Removing the hierarchy of school grades. | 11 |
| **Faculty development** |  |
| - Provide character-related faculty development programs so that professors can set an example in clinical practice. - Raising professors’ awareness of character education and developing character education competency: If medical professors have a biased perception of the character a doctor should have, students observe behaviors different from those learned in the curriculum in the classroom and on-the-job training, so there is a high risk of halving the effect of character education at the university. Therefore, it is required to derive character-related behaviors that match the mission and intentions of the university, and to educate the professors first. - To form a core professor group who can take charge of character education. It is necessary to select an interest group from among the existing professors to develop the professor, and to determine the appropriate achievements for the professor's activities in advance. - It would be good to have a sufficient understanding of character education and evaluation possibilities in character education for the professors so that it can be applied to student education. - Professor development is required first. (Management of evaluation qualifications?) - Recently, unlike the past, students' character qualities are showing individual, selfish, and hypocritical behaviors, so it is urgent to develop a curriculum and professors specialized in character education. | 6 |
| **Disciplinary measures** |  |
| - Establishment of self-purification system of medical groups: internal disciplinary measures. - It is also necessary to instruct students not to cheat in school exams, etc. - Student delegation decides on the student code of conduct. - Solutions? I think introspection is important. I think there should be a clear standard for what is a problem with character and there should be a program that follows it. | 4 |
| **Miscellaneous** |  |
| - The biggest task is to restore the overall character and ethical culture of society. When society recovers, it seems that it will be a motivator to recover character as well. - If necessary, it is also important to generate and provide timely and credible materials that can help undergraduate education in related societies (research groups) or institutions, rather than entrusting it to individual schools and professors in charge. - It will be possible if the Good Samaritan Law is enacted as the superordinate rule of all laws. - Orientation and student counseling is required | 4 |
| **Negative comments** |  |
| - Although medical schools are making an effort to educate the character-quality factor through the regular or non-regular curriculum, it seems difficult to evaluate whether the education is actually being done properly. There is always a question as to whether the original character that has already been formed during the growth period can be changed into college education. It seems that just because a medical school provides character education is different from changing a student's individual personality. However, the medical school should not neglect its efforts, and since there will be some areas for improvement, it will have to work in various ways. - There is very little we can do about character education in medical school. Character building is home education. | 2 |
